# Sustainable Utilization of Lignite Bottom Ash (LBA) and Bituminous Bottom Ash (BBA) for Ceramic Foam Production

**DOI:** 10.1021/acsomega.5c06610

**Published:** 2026-04-03

**Authors:** Wadwan Singhapong, Angkhana Jaroenworaluck, Watchara Chokevivat, Chutima Vanichvattanadecha

**Affiliations:** † National Metal and Materials Technology Center (MTEC), 61191 National Science and Technology Development Agency (NSTDA), 111 Thailand Science Park, Phahonyothin Road, Khlong Nueng, Khlong Luang, Pathum Thani, 12120, Thailand; ‡ National Nanotechnology Center (NANOTEC), National Science and Technology Development Agency (NSTDA), 111 Thailand Science Park, Phahonyothin Road, Khlong Nueng, Khlong Luang, Pathum Thani, 12120, Thailand

## Abstract

In this study, different sources of coal bottom ashes, industrial solid wastes/byproducts, generated from two coal-fired power plants, were utilized as raw materials in the ceramic industry due to their high contents of silica (SiO_2_). As-received lignite bottom ash (LBA) and bituminous bottom ash (BBA) were characterized to identify chemical compositions, present phases, particle size distributions, and microstructures by using the techniques of XRF, XRD, SEM, and particle size distribution, respectively. LBA and BBA were ground, dried, and mixed with a commercial alumina (Al_2_O_3_) with weight ratios (%) of the commercial alumina: the ground ashes as equal to 70:30 and 50:50, which were used to fabricate ceramic foams via a replica technique. Green foams were sintered in the range of 1250–1450 °C. The experimental results showed that the ground LBA and BBA could be used as raw materials for the ceramic industry. Different properties of phase transformation, sintering temperatures, and compressive strengths were investigated, evaluated, and discussed.

## Graphical abstract

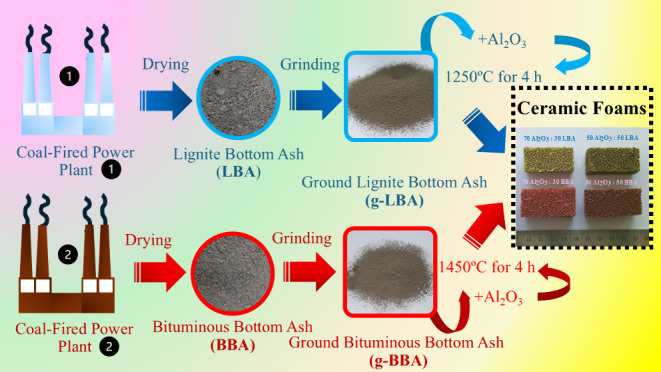

## 1 Introduction

Coal has been known as one of the natural resources utilized as energy fuels for producing electricity by combustion technology.^1–6^ Recently, the technology burning coal has been claimed as a source of producing a greenhouse effect, impacting air-polluted environments. Consequently, the use of coal has been monitored, and we are required to reduce its consumption before we turn to a new era of fully alternative energy productions. The Circular Economy (CE) model applied in European countries and other countries^7–10^ has been related to a main concept of recycling by turning waste into waste wealth or secondary raw materials. As globally known, coal-fired power plants have generated enormous amounts of industrial solid wastes/byproducts of coal fly ash (CFA), coal bottom ash (CBA), and flue gas desulfurization (FGD) gypsum from the coal combustion process.^1–3,11,12^ CBA has been considered following the CE involving the approaches of rare earth element (REE) extraction and waste recycling.^6,13^ As reported previously, the largest amount of the byproducts, generated from the coal-fired plant, is CFA.^3,5^ Amounts of CFA and CBA generated in each plant were varied in the range of 75–90% CFA:10–25% CBA,^2,3,14^ while the amount of the FGD gypsum would depend on the process of flue gas desulfurization, applied with the aim mainly to reduce sulfur dioxide (SO_2_) gas, reacting with calcium-based materials such as lime (CaCO_3_) to form calcium sulfate dihydrate (CaSO_4_·H_2_O).^12^ When compared to CFA and CBA, FGD gypsum known as an artificial gypsum, having the brown color, has been reported that it can be used generally in the construction industry because its composition is similar to natural gypsum.^11,12^ In addition, it can be applied for soil amendment.^11^ Oppositely, CFA and CBA could not be used directly as raw materials without mixing with other raw materials in practical applications. This is a reason why CFA and CBA have been used as sand replacement^5,15^ or aggregates in concrete.^5,16^

CFA and CBA collected from coal-fired plants have been generally characterized by using the X-ray fluorescence analysis (XRF) method to evaluate their oxide compositions. In general, the XRF analyzed results revealed the similar oxide trend, showing that both of them consist of the major oxide of silica (SiO_2_) and the 3 minor oxides of alumina (Al_2_O_3_), calcium oxide (CaO), and iron oxide (Fe_2_O_3_).^3–5^ However, CFA is mostly used in the cement industry because of its fine particles, having the benefits to general users to use it as a raw material or a filler/additive material mixed with cement to replace aggregates or sand in the mixtures of concrete due to its pozzolanic property.^17,18^ Although CBA has been revealed to have pozzolanic properties similar to those of CFA^19^ and could be used in mortar and cement as greener building materials in construction industries,^14,20^ the use of CBA is still limited because of inhomogeneous particle sizes, resulting in increased costs of the milling processes prior to use.^21,22^ This may be one of the reasons why CBA is still left in coal-fired power plants and needs to be found by end users. Obviously, CFAs have been used to produce many kinds of ceramic powders such as zeolite,^23,24^ mesoporous silica (SBA-15),^25,26^ etc. Furthermore, mullite, an advantageous ceramic powder in terms of heat resistance, could be prepared by mixing CFA with (i) alumina,^27^ (ii) aluminum dross industrial waste,^28^ and (iii) alumina with clay.^29^ Based on the similarity of the chemical compositions of CFA and CBA, recent studies have shown that CBA can be used in the same way as CFA as a raw material for the synthesis of zeolite,^3,30^ mesoporous silica,^3^ mesoporous-SBA-15,^31^ and other adsorbents.^3^

The aim of this present study is to seek a novel approach to utilizing CBA as much as possible as a raw material in the ceramic industry instead of using it in the cement and concrete industries mainly. In addition, this will extend the possibility of its usage in practical applications. CBA obtained from two coal-fired power plants where lignite and bituminous coals were used as the fuel sources for producing electricity were used to study whether both have a potential for further utilization. As-received CBAs were characterized, ground, and mixed with a commercially available alumina (Al_2_O_3_) powder to replace the raw material of silica or alumina, used in the fabrication process of ceramic foams, having the potential for further use as ceramic filters due to their 3-dimensional interconnected macropores. Significantly, the structure of the interconnected macropores of ceramic foams is beneficial for use in 2 typical industries of biomaterials as the bone replacement materials^32,33^ and casted metal parts as molten metal filters.^34,35^ Since CBA is still considered an industrial waste, it is difficult to use in the field of medical application. However, it is possible to use CBA to produce the ceramic foams fabricated in this work as ceramic foams for the applications of polluted water/air purification and energy applications in further.^36^

In this study, turning phase transformation into a mullite phase of the mixtures during the sintering process was investigated and discussed due to the valuable mullite being able to be used in a variety of ceramic applications.

## 2 Materials and Methods

### 2.1 Materials

Figure 1 shows the experimental procedure for this study. Lignite bottom ash (LBA) and bituminous bottom ash (BBA) powders were received from two different coal-fired power plants and stations located in different provinces of Thailand: Lampang province (Northern part) and Royong province (Eastern part). Commercial alumina powder (Al_2_O_3_) and silica (SiO_2_; S22, Belgium), used as control powders, were donated from RiO Tinto Alcan Inc., Canada and a laboratory, respectively.

**Figure 1:**
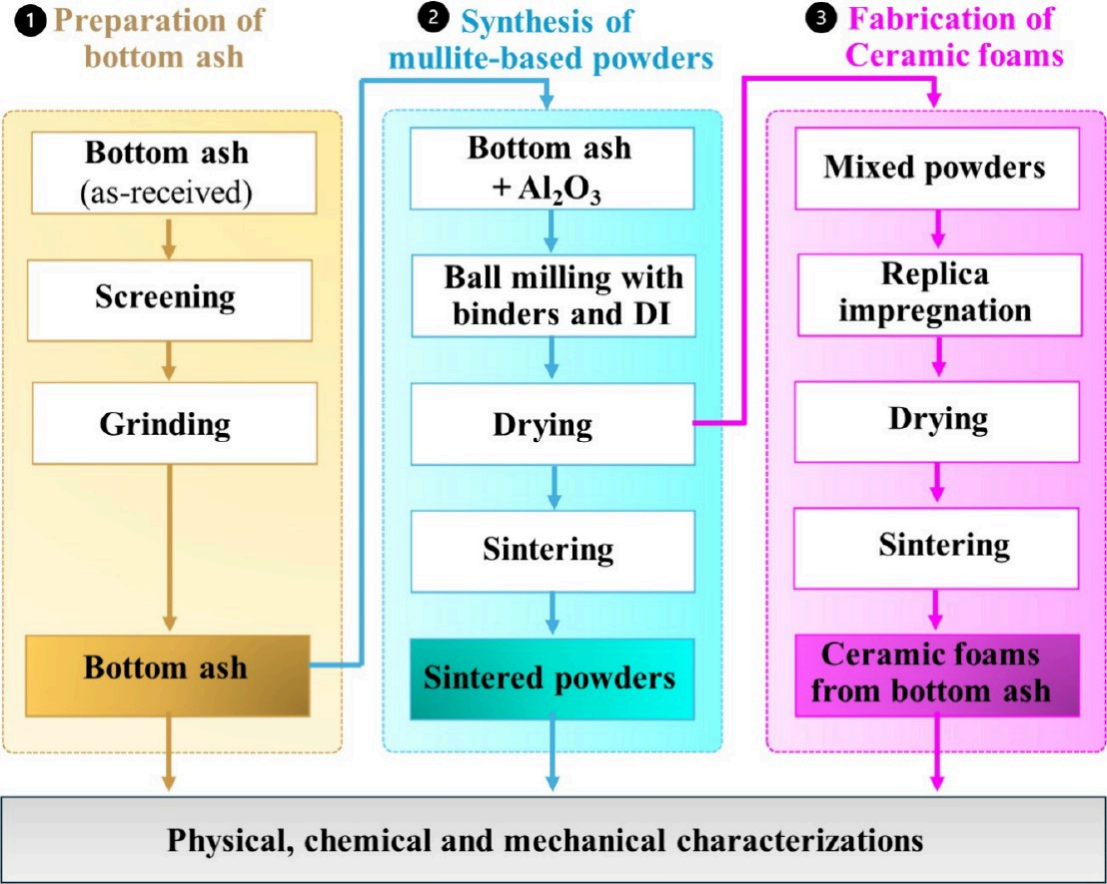
A flowchart of the experimental procedures used for this study.

Prior to use, as-received LBA and BBA powders were dried in an oven at 80 °C for 24 h. Amounts of moisture contents (W) contained in LBA and BBA powders were calculated by using the equation given below

$$\mathrm{Moisture content}\left(\%\right)=\left(W_{a}-W_{d}\right)/W_{a}\times 100$$ 1

where W_a_ is the weight of the as-received CBA or BBA and W_d_ is the weight of dried CBA or BBA.

### 2.2 Ceramic Processing

Based on visual observation, larger sizes (more than 1.0 cm) of the as-received LBA and BBA were taken out from the process of grinding. Both as-received LBA and BBA were ground by using a wet ball-milling method, in which plastic mills and zirconia balls were used as containers and the grinding media, respectively. The wet grinding process was continuously performed for 24 h. The balls were separated from the slips prior to drying in an electric oven at 80 °C until the slips were dried. Hereafter, LBA, BBA, g-LBA, and g-BBA are designated as-received LBA, as-received BBA, ground LBA, and ground BBA, respectively.

The dried LBA and BBA were mixed with a commercially available alumina (Al_2_O_3_) with weight ratios of A_2_O_3_: g-LBA or g-BBA as 70:30, 50:50. The mixtures were mixed by using the wet ball-milling process for 1 day as same as the grinding process and finally dried in the oven at 80 °C. The dry mixtures were further used for fabrication of ceramic foams as in a method reported in a previous study.^37^ In this present study, 20 ppi of a type of polymer foams were cut into approximately 50 × 25 × 20 mm used as the macropore templates, to determine a proper sintering temperature and fabrication conditions by sintering at maximum temperatures of 1250, 1300, 1400, and 1450 °C for 4–8 h. Finally, 7 ppi of the polymer foams were used as the pore templates for increasing macropore sizes compared with final products of commercial foams used as ceramic foam filters.

### 2.3 Characterizations and Mechanical Tests

Both dried LBA and BBA were characterized prior to use. In addition, in this study, other raw materials (lignite coal (LC), bituminous coal (BC), lignite fly ash (LFA), and bituminous fly ash (BFA)) were requested from both plants to identify the characteristic differences of each coal and ash. Chemical compositions and total carbon contents of each sample were characterized by a wavelength-dispersive X-ray fluorescence spectrometer (WDXRF; ZSX Primus, Rigaku, Japan) and a total organic carbon analyzer (TOC; SSM-5000A, Shimadzu, Japan), respectively.

Particle size and microstructures of as-received LBA and BBA were characterized by a field emission scanning electron microscope (FE-SEM; HITACHI SU5000, Japan). Particle size distributions were also determined with an analyzer (Mastersizer 2000, Malvern, UK). The phase presence of all samples was identified by an X-ray diffractometer (XRD; TTRAX III, Rigaku, Japan) operating at Cu–K_a_ radiation with 40 kV and 30 mA. All the samples were scanned in the range of 5–80° 2θ with a fixed scan speed of 2.4° 2θ/min.

To perform the compressive strength tests, a set of samples (4 samples per each batch composition) were prepared by using 7 ppi of the polymer foams having their cut sizes of approximately 60 mm × 40 mm × 30 mm. The 7 ppi foams were the largest macropore size of the polymer templates used in this study. Green samples were finally sintered at 1250 °C for 4 h and 1450 °C for 4 h for the batch compositions of LBA and BBA, respectively. The compressive strengths of all sintered samples were measured at a loading speed of 0.1 mm/min using a universal tester (EZ-LX, Shimazu, Japan) connected with the Texture analyzer software. One kN of a load cell was used in this study.

### 2.4 MTT Cytotoxicity Assay

#### 2.4.1 Sample Preparation

The MTT assay test performed in this study was illustrated as shown in Figure 2. For the extraction ratio, 0.1 g/mL of each sample was added into separated centrifuge tubes (50 mL) and sterilized by using an autoclave (Hirayama HVE-50, Japan) at 121 °C for 15 min. The samples were then mixed with the minimum essential medium (MEM, Lonza, USA), the completed medium, containing 10% horse serum (Gibco, USA), 1% Pennicillin/Streptomycin (Gibco, USA), and 1% GlutaMax (Gibco, USA). Finally, the samples were extracted by using a roller mixer (Stuart Scientific, SRT2, UK) at 37 ± 1 °C for 24–26 h.

**Figure 2:**
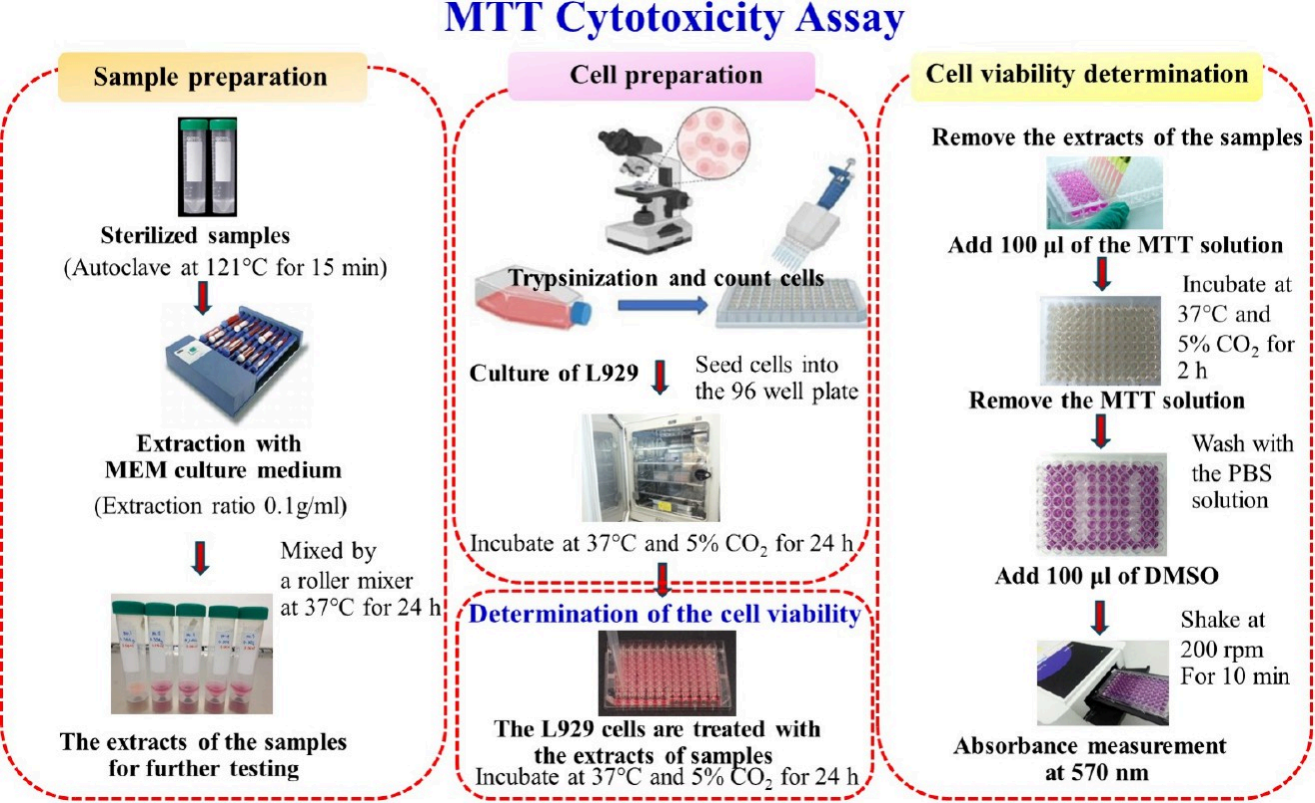
An illustration showing experimental procedures for the MTT assay test performed in this study.

#### 2.4.2 Cell Preparation

Mouse fibroblast cells (ATCC, USA) of strain L (CCL1, NCTC 929) were used in this study. Prior to use, the cells were checked using a phase-contrast microscope (Leica, DMIL, Germany). The phosphate buffer saline (PBS) buffer solution was used to wash the solution adhered on the cells after the medium was taken out. For cell dissociation, trypsinization was carried out by using Trypsin-versene (Lonza, USA), a proteolytic enzyme to dissociate adherent cells from the vessel in which they were cultured as cell suspensions by adjusting cell concentrations 1 × 10^5^ cells/ml. The cell suspensions (200 μL) were added into each well of 96-well plates using multichannel pipettes and incubated in a CO_2_ incubator (Forma Scientific, Forma 3111, USA) at 37 ± 1 °C for 24 ± 2h, with conditions of 5 ± 1% CO_2_, 95 ± 5% RH (Relative humidity).

#### 2.4.3 Viability Determination

Discard the medium from 96-well plates, add the extracts from the samples and the controls and incubate them in a CO_2_ incubator for 24–26 h. 0.5 mg/mL of the MTT solutions ((3-(4,5-Dimethylthiazol-2-yl)-2,5-Diphenyltetrazolium bromide); Sigma-Aldrich, USA) was prepared, and 100 μL of the prepared MTT solutions was added into each well and incubated 2 h. The cells were then washed with the PBS solution. 100 μL of dimethyl sulfoxide (DMSO; Sigma-Aldrich, USA) was added into each well and shaken using a shaker (Shaker; GFL, Germany) at 200 rpm for 10 min. The absorbance or optical density (OD) of the extract was measured at 570 nm by using a microplate reader (Biochrom Easys, UVM 340, USA). The mean cell viability (%) was expressed as shown in eq 2 ^38^

$$\mathrm{Cell viability}\left(\%\right)=\left({\mathrm{OD}}_{570e}/{\mathrm{OD}}_{570b}\right)\times 100$$ 2

where OD_570e_ is the mean value of the measured optical density of the 100% extracts of the test sample and OD_570b_ is the mean value of the measured optical density of the 100% extracts of the blank.

## 3 Results and Discussion

### 3.1 Typical Characteristics of the Ashes

#### 3.1.1 Visual Characteristics

Figure 3a,b (on the left side) reveals the characteristics of LBA and BBA obtained from the electricity plants, respectively. After receiving the ashes, all ashes were immediately dried in ovens to prevent microbial formation from the moist contents. Glassy-melted particles having big sizes (approximately 10–30 mm) mixed in the as-received LBA could be seen easily after drying as yellow-circled in the right photograph of Figure 3a. By visual inspection, it is difficult to distinguish the difference between LBA and BBA. However, LBA had a lot of bigger size particles (>10 mm) when compared with those of BBA. After drying, slight color differences could be seen. The color of LBA was changed from black into the gray-black tone while the color of the dried BBA was turned into the red-black tone. LBA particles had more variation in particle sizes when compared to that of the BBA. This may be related to the different grinding methods of the coal used as the fuel in the combustion process of each coal-fired power plant. Both LBA and BBA were received in wet conditions from both plants. The different moisture contents, as shown in the inserted table of the figure, may come from the water spray employed to suppress airborne particles before transporting the ashes from the combustion unit to the land fill areas of each plant.

**Figure 3:**
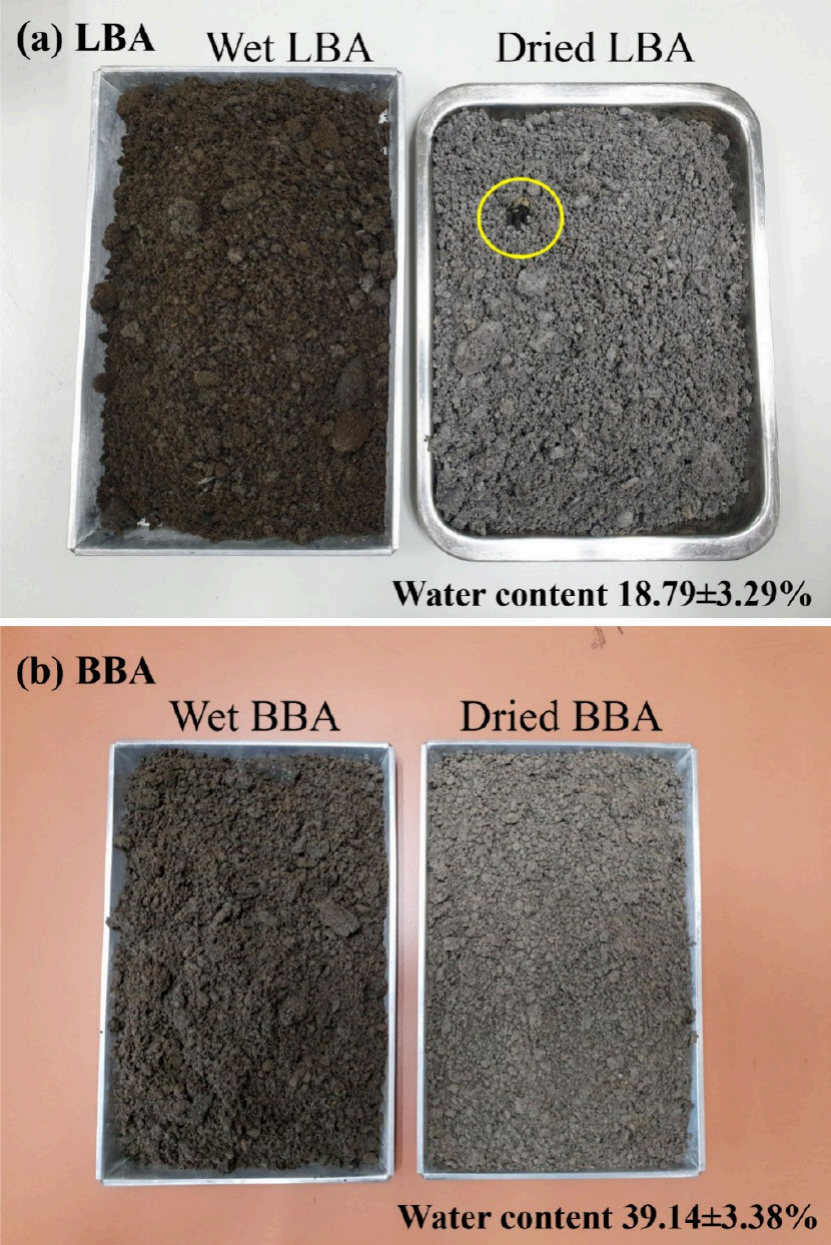
As-received LBA (a) and BBA (b) before and after drying.

#### 3.1.2 Chemical Compositions

Table 1 reveals a significant characteristic of chemical compositions of LBA and BBA compared with LFA, BFA, LC, and BC. Similarly, both LBA and BBA consist of silica (SiO_2_) as the highest oxide amount, followed by alumina (Al_2_O_3_), calcium oxide (CaO), and iron oxide (Fe_2_O_3_). Other oxides were also found, but significant amounts could not be seen. However, LBA showed that the amount of CaO was higher than that of BBA. Obviously, sulfur oxide (SO_3_) contained in LBA was also higher than that of BBA. This finding is the same tendency of LFA compared with BFA. Significantly, boron oxide (B_2_O_3_) was found only in the LFA. Previous studies reported that boron oxide needed to be leached out to meet the standard regulation.^39,40^

**Table 1:** Chemical Compositions of Coal, Bottom Ashes, and Fly Ashes Analyzed by Using XRF Analysis

	**Lignite Bottom Ash**	**Bituminous Bottom Ash**	**Lignite Fly Ash**	**Bituminous Fly ash**	**Lignite Coal**	**Bituminous Coal**
**Oxides (%)**	**(LBA)**	**(BBA)**	**(LFA)**	**(BFA)**	**(LC)**	**(BC)**
B_2_O_3_			4.1225			
Na_2_O	1.0209	1.6921	1.7648	3.1773	0.2827	0.0759
MgO	2.1071	1.9430	2.5576	3.0027	0.5714	0.1203
Al_2_O_3_	11.4050	17.6786	10.3076	22.0416	3.4357	0.8213
SiO_2_	23.4249	56.4959	20.9899	46.8235	5.9473	1.5995
P_2_O_5_	0.2221	0.1624	0.3116	0.3183	0.0441	0.0100
SO_3_	5.0494	0.2076	6.0283	2.2000	6.2353	1.0356
K_2_O	2.1351	1.4430	1.7375	1.7143	0.3763	0.0566
CaO	29.8706	5.5341	30.7823	7.0406	5.7583	0.2094
TiO_2_	0.4701	0.9896	0.3521	1.1128	0.0732	0.0236
Cr_2_O_3_	0.0203	0.0183	1.3979	0.0876	0.0041	
Fe_2_O_3_	21.6441	11.6985	18.5490	11.5061	2.7795	0.3000
SrO	0.2382	0.1420	0.1764	0.1538	0.0262	0.0029
LOI	2.06	1.69	0.60	0.57	74.43	95.74
Total	100.00	100.00	100.00	100.00	100.00	100.00

In the cement and concrete industry, generally, coal fly ash (CFA) can be used as a filler or a raw material to mix with cement for increasing physical properties and controlling setting time.^41^ This can imply that CFA can partially replace the use of cement mixed with the concrete mixtures. In addition, they can be used by replacing aggregates or sand. Oxides contained in LC and BC are related to the total amount of loss of ignition (LOI), resulting from the total amounts of carbon in LC and BC before heating/firing. As presented in the table, BC had a higher LOI content than that of LC.

As shown in Table 2, when considering all important oxides used in the ceramic industries, it can focus only on the amounts of SiO_2_, Al_2_O_3_, CaO, Fe_2_O_3_, and SO_3_. It is found that BBA had higher amounts of SiO_2_ and Al_2_O_3_ than those oxides in LBA. Furthermore, higher contents of CaO, Fe_2_O_3_, and SO_3_ in LBA are significantly observed. For the cement industry, according to ASTM C618 (Standard specification for coal fly ash and raw or calcined natural pozzolan for use in concrete),^5^ LBA and BBA used in this study can be classified as Class C (SiO_2_ + Al_2_O_3_ + Fe_2_O_3_ ∼ 50–70 wt % with the high amounts of CaO) and Class F (SiO_2_ + Al_2_O_3_ + Fe_2_O_3_ ≥ 70 wt % with the low amounts of CaO), respectively.^3–5^ It is obvious that Classes C and F can also be designated for both the as-received LFA and BFA, respectively. Differences in the main oxides of each ash are correlated to the original compositions in lignite and bituminous coals.^3^ However, for the ceramic industry, it has been known that calcium oxide and iron oxide can be served as sintering aids or fluxing agents by mixing one of these oxides or both with other raw materials to reduce sintering temperatures for firing the final products of ceramics.^7,42,43^ When sintering the green bodies or glazes mixed with the iron oxide, the sintered products will have brown tone colors.^44^ Interestingly, SO_3_ present in the ashes is typically chemically combined with other oxides to form sulfates, most notably anhydrite (CaSO_4_). It can act as fluxing agents during sintering as well.^45^

**Table 2:** Comparison of Typical Oxides from the XRF Results Derived from Table 1

	**Oxides (%)**								
**Samples**	**SiO**_**2**_	**Al**_**2**_ **O**_**3**_	**Fe**_**2**_ **O**_**3**_	**SiO**_**2**_ **/Al**_**2**_ **O**_**3**_	**SiO**_**2**_ **+Al**_**2**_ **O**_**3**_	**SiO**_**2**_ **+Al**_**2**_ **O**_**3**_ **+Fe**_**2**_ **O**_**3**_	**CaO**	**SO**_**3**_	**LOI**
LBA	23.4249	11.4050	21.6441	3.8943	34.8299	54.474	29.8706	5.0494	2.06
BBA	56.4959	17.6786	11.6985	3.1957	74.1745	85.873	5.5341	0.2076	1.69
LFA	20.9899	10.3076	18.5490	2.03635	31.2975	49.8465	30.7823	6.0283	0.60
BFA	46.8235	22.0416	11.5061	2.1243	68.8651	80.3712	7.0406	2.2000	0.57
LC	5.9473	3.4357	2.7795	1.7310	9.383	12.1625	5.7583	6.2353	74.43
BC	1.5995	0.8213	0.3	1.9475	2.4208	2.7208	0.2094	1.0356	95.74

Table 3 shows results of the evaluation of total carbon in each sample determined by oxidizing inorganic and organic carbon at high temperature, resulting in the release of carbon dioxide (CO_2_). The carbon contents were quantified based on the measured CO_2_ level. As shown in the table, bituminous coal (BC) had a higher total carbon (TC) than that of lignite coal (LC). However, after firing the coals, the amount of TC of BBA was less than that of LBA. This finding can imply that BBA has almost complete firing conditions when compared to LBA, turning into loss of ignition (LOI). Due to the presence of TC in LFA, the color of LFA was darker brown than that of BFA. However, the color of BFA ashes was in color tones of pale brown to brown. This may relate to the high amounts of chemical compositions in each ash. The ashes containing higher carbon contents would have darker brown-black color tones of brown-black. Based on the results found in the table, carbon contents in as-received ashes after coal-firing processes affect their color appearances.

**Table 3:** Total Carbon (TC), Total Organic Carbon (TOC), and Total Inorganic Carbon (TIC) of Coals (Cs), Coal Bottom Ashes (CBAs), and Coal Fly Ashes (CFAs)

	**Total Carbon (TC)**	**Total Organic Carbon (TOC)**	**Total Inorganic Carbon (TIC)**
**Samples**	(%)	(%)	(%)
Lignite coal (LC)	39.80	39.18	0.62
Bituminous coal (BC)	57.83	57.83	0.00
Lignite bottom ash (LBA)	0.62	0.42	0.20
Bituminous bottom ash (BBA)	0.27	0.27	0.00
Lignite fly ash (LFA)	0.19	0.03	0.16
Bituminous fly ash (BFA)	0	0	0

#### 3.1.3 Particle Size Distributions

Particle size distributions of all the as-received ashes (CBA and CFA) were measured by the technique of laser diffraction analysis using water as the dispersant media. Figure 4 shows the analyzed results. Particle size distributions of both LBA and BBA were unimodal characteristics. The average particles size of LBA and BBA were 145.71 and 90.02 μm (D_50_), respectively, whereas the profiles of LFA and BFA were bimodal distributions. Significantly, both as-received fly ashes were smaller than those of the as-received bottom ashes. The first and second ranges of the distributions of LFA and BFA were 0.5–2.0 and 2–300 μm. The average particle sizes of LFA and BFA were 11.55 and 8.19 μm (D_50_), respectively. The analyzed analysis showed that LFA and BFA received from the plant were fine particles, and no further grinding process was required prior to the analysis. To utilize LBA and BBA as ceramic raw materials, a grinding process is required to reduce their particle sizes prior to performing the ceramic fabrication. Furthermore, reducing the particle sizes is a parameter that can lower the sintering temperatures of green samples.

**Figure 4:**
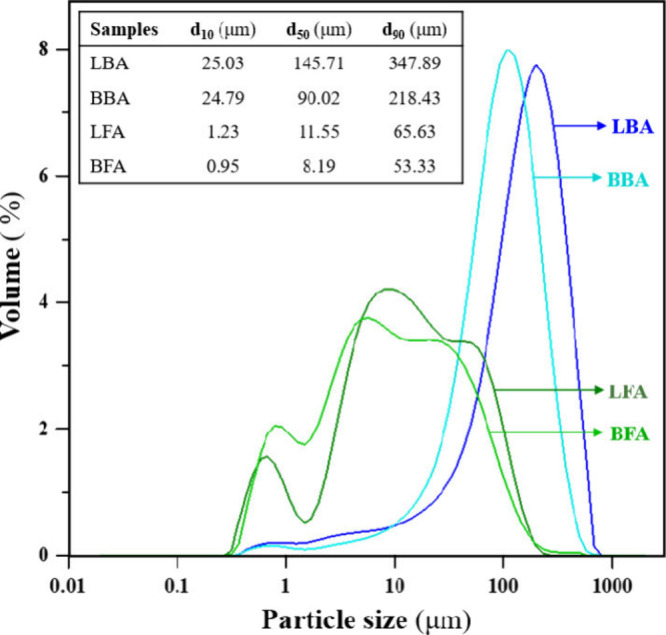
Particle size distributions of CBA and CFA obtained from different coal-fired power plants.

#### 3.1.4 Phase Presence

Figure 5 shows a comparison set of XRD profiles of LBA and BBA, compared with LFA and BFA. Due to the XRD techniques, as-received LBA and BBA were grounded by using the dry milling method to prepare the XRD samples. The XRD profile of LBA reveals the highest-intensity peak of calcium magnesium silicate, followed by the lower-intensity peaks of augite and anorthite, respectively. Minor intensity peaks of quartz, magnetite, and mullite could be found. For the XRD profile of BBA, only 3 main peaks could be observed. The highest peak was quartz, followed by the peaks of mullite and anorthite, respectively. It is interesting that LFA has more peaks of crystalline materials than those of BFA. Significantly, the profiles of LFA consisted of more various oxides than those of BFA as shown in the figure. The oxide peaks found in ashes may be correlated with the oxides in the coals. For BFA, it consists of only 3 phases of quartz, mullite, and anhydrite.

**Figure 5:**
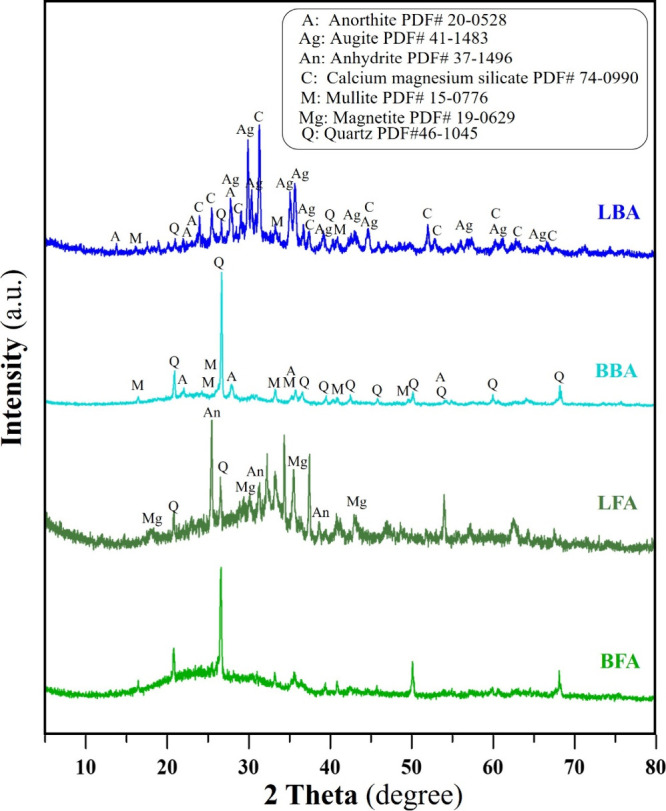
XRD patterns of dried as-received LBA and BBA compared with as-received lignite and bituminous fly ashes.

#### 3.1.5 Microstructure Observations

Figure 6a-d shows SEM images of LBA, BBA, g-LBA, and g-BBA. Figure 6e,f shows digital photographs of g-LBA and g-BBA, respectively. Colors of LBA and BBA were similar. In addition, grinding the ashes still had indifferences in color appearance (Figure 6e,f). Furthermore, LBA and BBA had similar SEM microstructures of agglomerated irregular particles with sharp edge surfaces. Some of the spherical particles the same as found in CFA could be seen.^46^ The spherical particles may be the particles in CFA that fall down and are mixed with the irregular particles of CBA. The agglomerated particles may result from incomplete combustion during the coal burning process. It is obvious that there are more rod shaped-particles in LBA than those of BBA. However, when grinding LBA and BBA, the glassy particles disappeared as shown in Figure 6e,f, showing the similarities of g-LBA and g-BBA in terms of the fine particles having brown-gray color shades.

**Figure 6:**
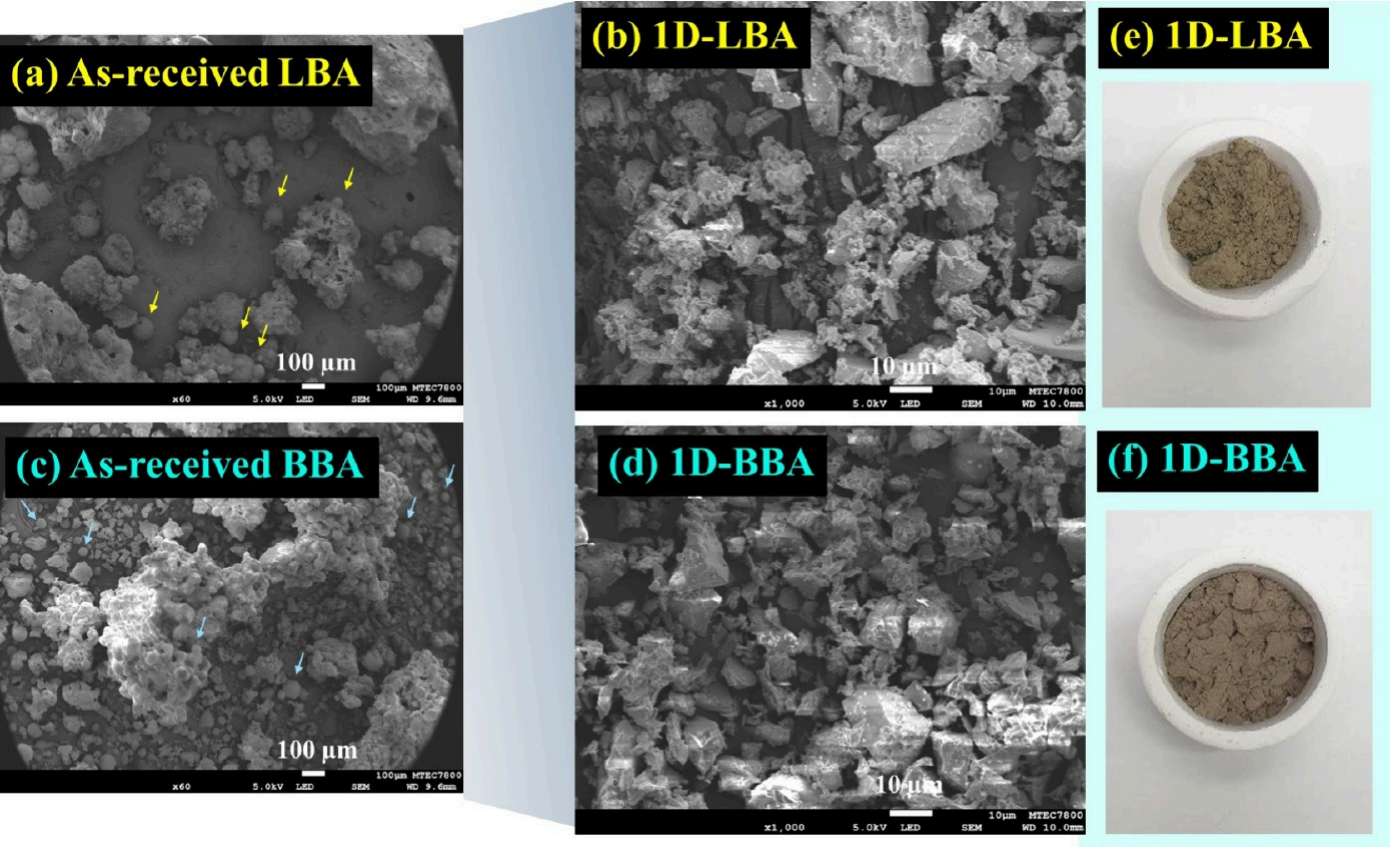
SEM images: (a,c) as-received LBA and as-received BBA, (b,d) as-received LBA and as-received BBA ground by the wet method for 1 day (24 h). Digital photographs of (e) and (f) the ground LBA and BBA for 1 day.

Figure 7a,c shows SEM images of LFA and BFA, respectively. Figure 7b,d are SEM images of LFA and BFA taken at higher magnified scales, respectively. The images reveal that both ashes are very fine particles. The microstructures of both ashes are similar, consisting of various sizes of the spheres as reported in the previous study.^24^ Based on the SEM observation, it is difficult to identify the hollow particles (open porous spheres) of Cenospheres^2,46^ since each of the spheres was complete in spherical shape particles without having the broken characteristics. Cenospheres mainly consist of SiO_2_ and Al_2_O_3_ filled with air/gases, resulting in floating on the water surface.^2,46^ Figure 7e,f shows color characteristics of LFA and BFA, respectively. The colors of LFA and BFA were in dark brown and brown tones, respectively. The different color appearances of LFA and BBA may relate to the carbon content left after the coal combustion process (see Table 3).

**Figure 7:**
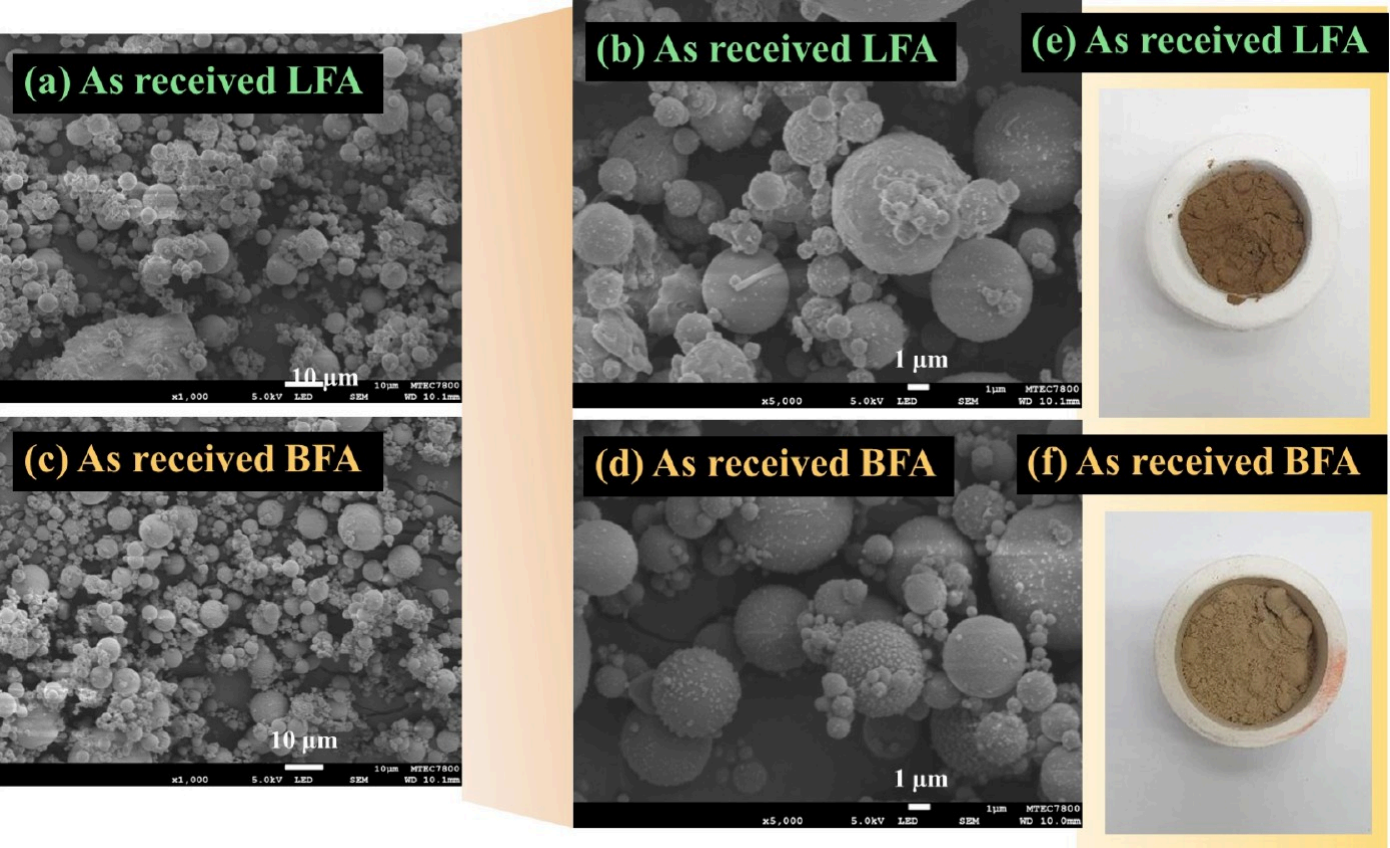
SEM images showing the microstructures of LFA (a)-(b) and BFA (c)-(d). Digital photographs of LFA (e) and BFA (f).

### 3.2 Determination of Sintering Temperatures

Table 4 shows the powdered formula used for fabricating ceramic foams using g-LBA and g-BBA as raw materials to mix with the commercial alumina. WF1 was used as a formula control prepared by mixing both commercial alumina and silica. The commercial silica was replaced by using g-LBA and g-BBA as shown in the table (WF2-WF5). The fabricated foams were finally sintered at maximum temperatures from 1250 to 1450 °C for 4 and 8 h. Figure 8a-e shows that all sintered foams had white color and the color did not change even though the sintering temperatures were increased. It also confirmed that the fabrication process could hold the shapes of the polymer foams used as pore templates even though the lowest sintering temperature was done at 1250 °C for 4 h (Figure 8a) and sizes of the sintered foams were smaller when sintering temperature increased (Figure 8a-e). As shown in Figure 8f, Al_2_O_3_ and SiO_2_ transform into corundum and cristobalite at 1250 °C, respectively.^47^ However, this temperature is insufficient to initiate mullitization. The formation of the mullite phase (3Al_2_O_3_·2SiO_2_) was identified by a low-intensity diffraction peak at 15° 2θ, which first appeared in the foams sintered at 1400 °C for 4 h. The intensity of the mullite peak increased in the foams sintered at 1450 °C for 4 and 8 h, coinciding with a significant decrease in the corundum and cristobalite phases. The presence of both residual phases suggests that the mullitization remains incomplete even after sintering at 1450 °C for 8 h.

**Table 4:** Powdered Formula for Ceramic Foam Fabrication

**Sample codes**	**Compositions** (wt %)
**WF1**	72% Al_2_O_3_ + 28% SiO_2_
**WF2**	70% Al_2_O_3_ + 30% g-LBA
**WF3**	50% Al_2_O_3_ + 50% g-LBA
**WF4**	70% Al_2_O_3_ + 30% g-BBA
**WF5**	50% Al_2_O_3_ + 50% g-BBA

**Figure 8:**
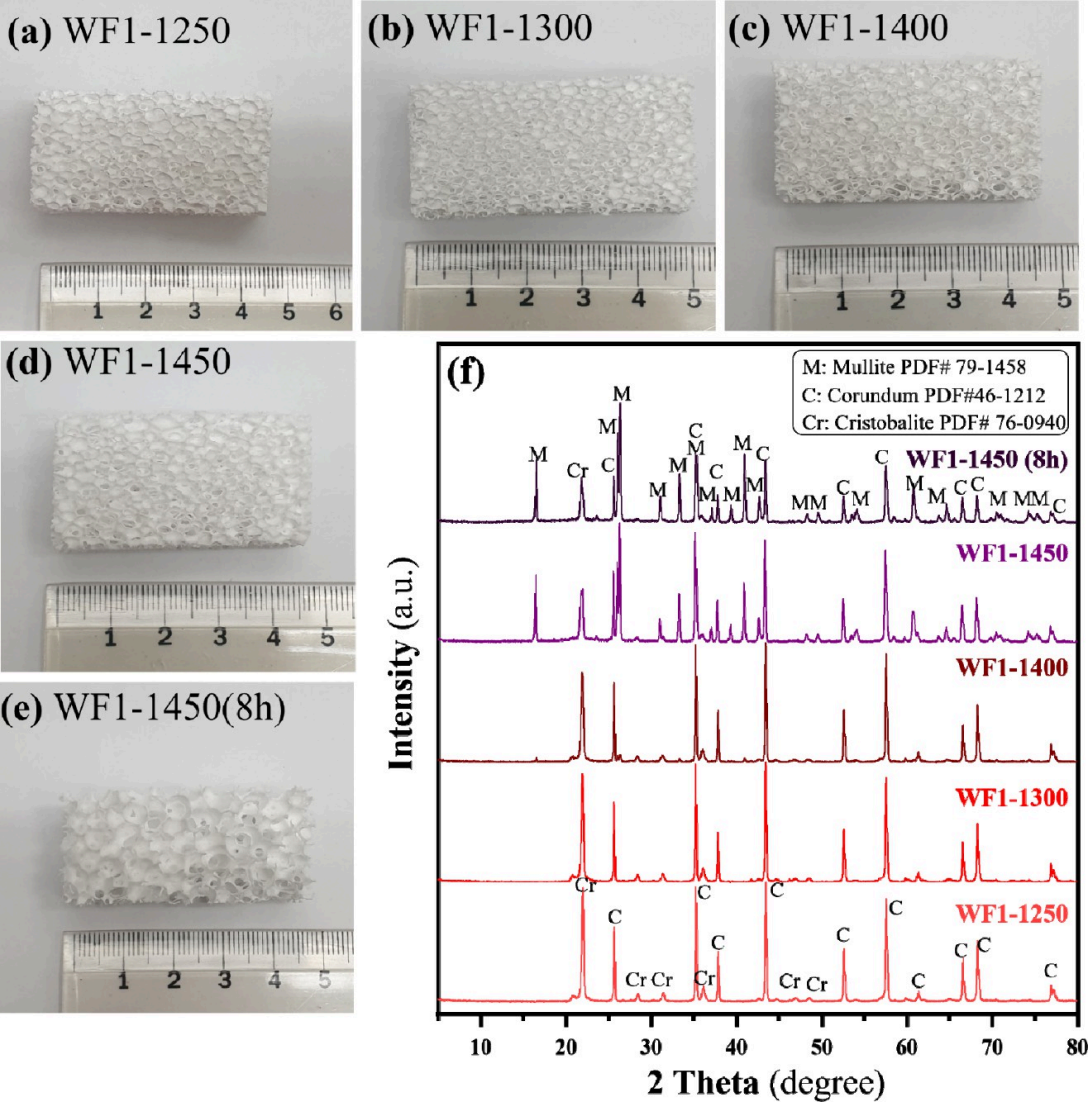
Fabricated foams used as control foams (WF1) sintered at (a)-(d) 1250, 1300, 1400, 1450 °C for 4 h and (e) 1450 °C for 8 h, respectively, corresponding XRD profiles of the sintered foams (f).

Figure 9a-1, a-2, b-1, and b-2 are photographs of sintered foams prepared by the formula WF2 and WF3 using g-LBA 30 and 50 wt % (see Table 4) in the formula, respectively. Based on the visual observation on the sintered samples as shown in the figure, it is found that the sintering temperatures were lower than that of the control foams (WF1), as evidenced by the foam shrinkage and collapse characteristics. When the foams were sintered at 1250 and 1300 °C for 4 h, the collapsed shapes of the sintered foams at 1300 °C for 4 h could be clearly found. This evidence directly implies that the sintering temperatures of the foams utilizing g-LBA should be less than 1300 °C and depend on the amounts of g-LBA mixed within the compositions. The colors of the sintered foams were also significantly related depending on the amount of g-LBA mixed with Al_2_O_3_. The WF2 formula added low amounts of g-LBA (WF2) had a green-orange color, whereas for high amounts of g-LBA (WF3), the color of the sintered foams was darkened in green tones as shown in Figure 9.

**Figure 9:**
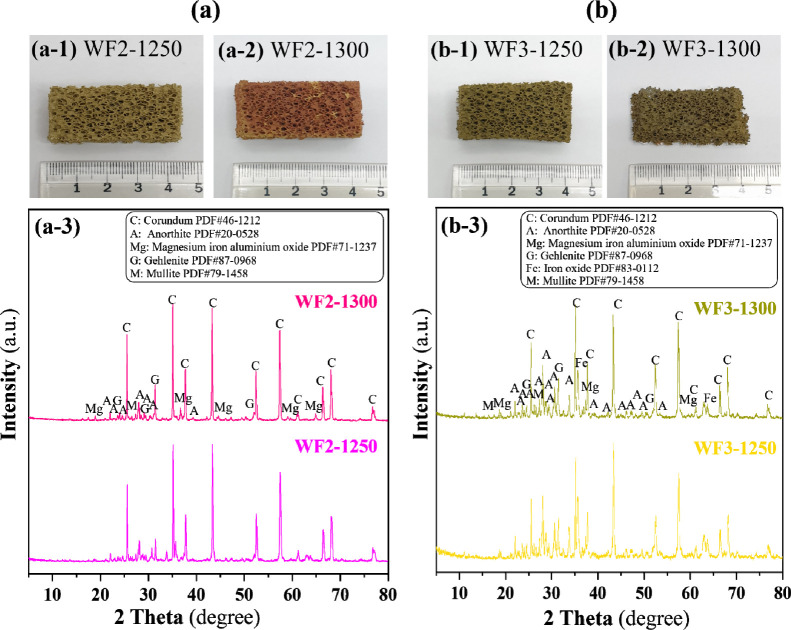
Digital photographs and XRD profiles of sintered foams from the mixtures of g-LBA and the commercial alumina with different weight ratios (WF2 and WF3) and sintered at maximum temperatures of 1250 and 1300 °C for 4 h, respectively.

Figure 9a-3 and b-3 show a comparison set of XRD profiles of WF2 and WF3 sintered at 1250 and 1300 °C for 4 h, respectively. XRD profiles of WF2 sintered at 1250 °C consisted of phases of corundum, mullite, gehlenite (Ca_2_Al­(AlSiO_7_), and anorthite (CaAl_2_Si_2_O_8_). While corundum and mullite arise from the phase transformation of Al_2_O_3_ and the mullitization process, respectively, gehlenite appears as a new phase absent in the as-received LBA. At high temperature, CaO reacts with aluminosilicate components in LBA to form gehlenite as an intermediate phase, which can further transform into anorthite.^48–50^ The coexistence of gehlenite and anorthite implies an incomplete transformation; this is likely due to the presence of residual mullite and quartz in the LBA, which may inhibit the complete transition of gehlenite.^49,51^ For XRD profiles of WF2 sintered at 1300 °C, peaks of magnesium iron aluminum oxide could be seen. This phase may be related to the color difference in each sintered foam using g-LBA in the formula.

Figure 10a and b are digital photographs of a set of WF4 foams compared with a set of WF5 foams sintered at maximum temperatures of 1250–1450 °C for 4 h (a-d) and for 8 h (e), respectively. The photographs reveal clearly that the color and shrinkages of the sintered foams were related to (i) the formula having higher amounts of g-BBA (WF5) in the formulas (ii) sintering temperature and (iii) holding sintering time which could promote the deeper color shades of brown color. The brown colors of samples may be related to the impurity iron oxide in CBA. In addition, the maximum sintering temperature of the foams using g-BBA should be in a range of 1450 °C.

**Figure 10:**
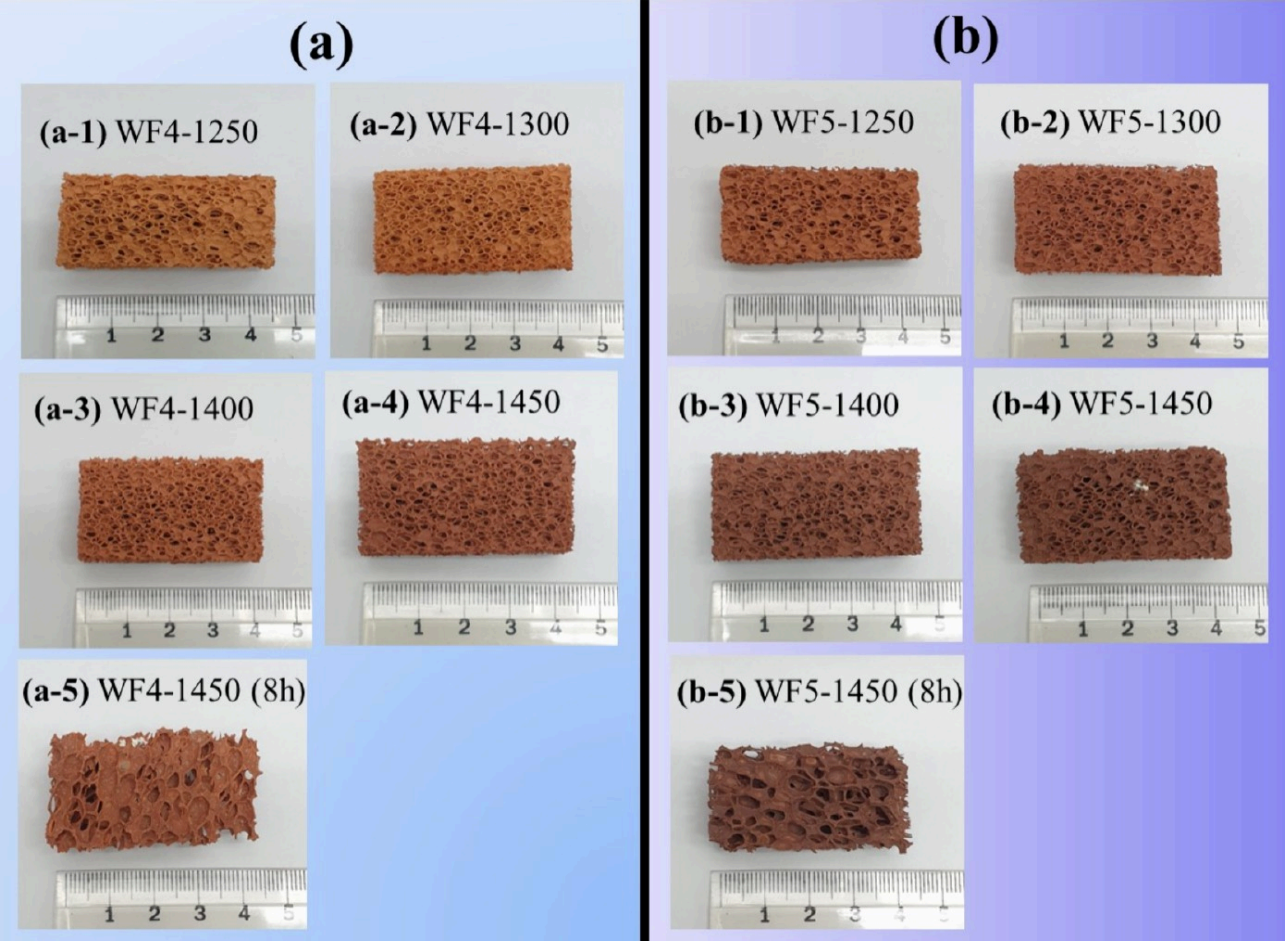
Digital photographs of the foams: (a) WF4 and (b) WF5 sintered at maximum temperatures of 1250–1450 °C for 4 h and 1450 °C for 8 h, respectively.

Figure 11a and b are XRD profiles of the WF4 and WF5 foams sintered from 1250 to 1450 °C for 4 and 8 h. The profiles reveal the partial transformation of SiO_2_ and Al_2_O_3_ phases into the mullite phase, impacting the detection of the phase transformation of corundum and cristobalite phases of SiO_2_ and Al_2_O_3_. Significantly, higher amounts of g-BBA mixed in the WF5 formula showed higher amounts of the mullite phase formed, resulting in a high-intensity peak of the mullite phase that could be seen when comparing XRD profiles shown in Figure 11a,b. The difference in maximum sintering temperatures between the foams derived from g-LBA and those from BBA is likely attributed to compositional differences. Particularly, the LBA contains higher contents of Fe_2_O_3_, CaO, and SO_3_ than those of BBA, according to the XRF results shown in Table 1. These oxides function as fluxing agents that promote the liquid phase formation, thereby lowering the deformation temperature and inducing structural collapse.^52^ In contrast, the BBA-derived foams are rich in Al_2_O_3_ and SiO_2_, which facilitate the formation of mullite—a phase that remains stable at very high temperature.

**Figure 11:**
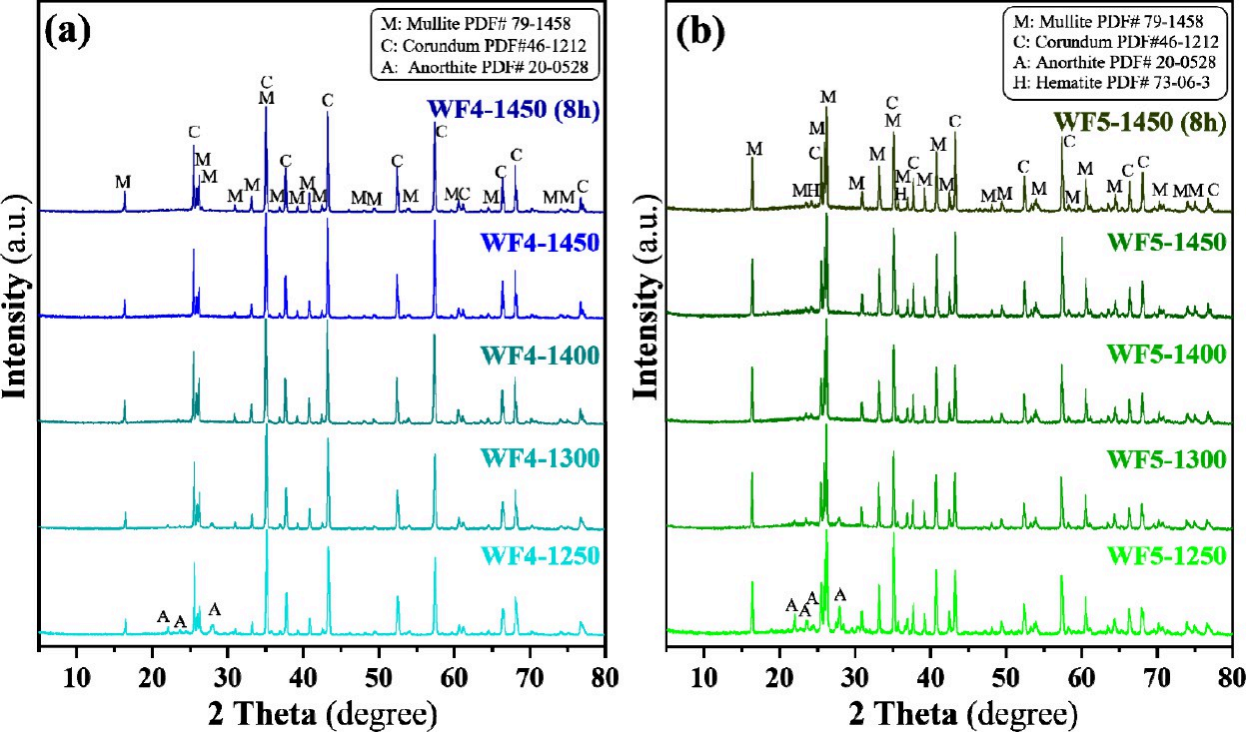
XRD profiles of the foams: (a) WF4 and (b) WF5 sintered at maximum temperatures of 1250–1450 °C for 4 and 8 h.

### 3.3 Mechanical Properties of Sintered Foams

Figure 12a presents the compressive stress–strain curves of the sintered foams derived from LBA sintered at 1250 °C for 4 h and BBA sintered at 1450 °C for 4 h. All of the foams exhibit typical deformation behavior of highly porous ceramics, beginning with a linear elastic region followed by a sudden stress drop corresponding to crack initiation at the surface. However, its high pore volume prevents crack propagation, leading to a plateau region associated with progressive fracture of the foam skeleton.Upon further compression, densification occurs, revealed by an increase in stress.^53^ Figure 12b indicates that all WF foams display similar stress–strain profiles, with average compressive strengths of 0.12, 0.15, and 0.14 MPa for WF2, WF3, and WF5, respectively. The highest strength, 0.22 MPa, was observed in WF4 derived from BBA (70 wt % Al_2_O_3_ + 30 wt % BBA). However, the difference among samples is not significant, indicating less compositional influence on the strength at this sintering temperature. Compared with commercial alumina foams with 10 ppi (∼0.8 MPa)^54^ and reticulated Al_2_O_3_ foams with 10 ppi (∼0.4 MPa),^55^ the sintered foams fabricated in this study exhibit slightly lower strength. However, this difference likely arises from variations in the pore sizes, pore volumes, and foam densities. Increasing the sintering temperature may enhance the development of phase transformation, particularly the mullite phase formation, thereby improving the mechanical strength.

**Figure 12:**
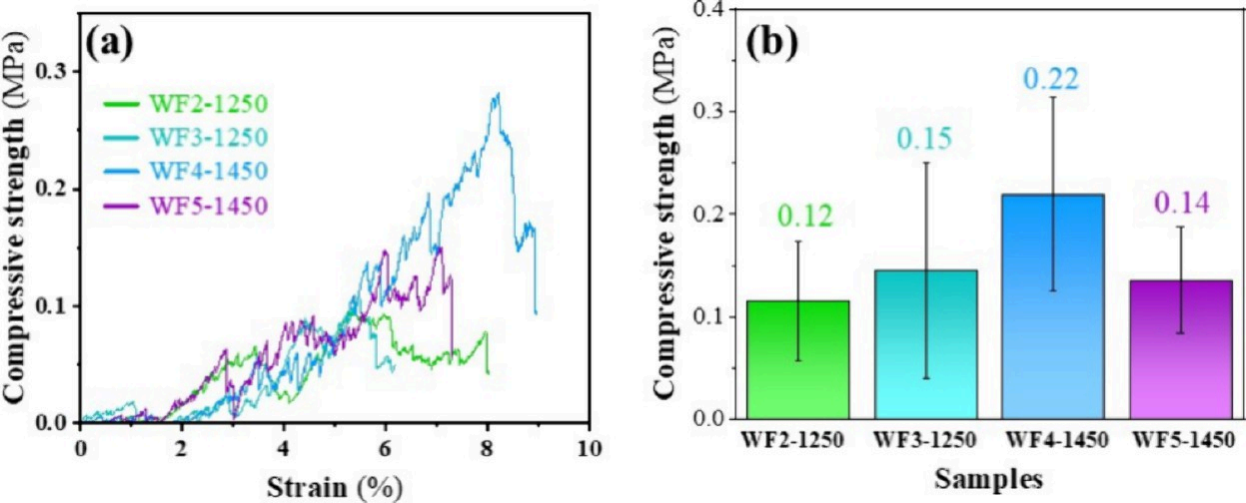
Compressive test results: (a) a set of samples showing their stress–strain curves and (b) compressive strength values of the sintered foams.

### 3.4 MTT Cytotoxicity Results

The cytotoxicity of g-LBA, g-BBA, and the foams (WF1-WF5) sintered at 1250 °C for 4 h°C was evaluated by MTT assay, a colorimetric technique to evaluate viable cells. The yellow tetrazolium salts of 3-(4,5-Dimethylthiazol-2-yl)-2,5-Diphenyltetrazolium bromide (MTT) were reduced to form the insoluble purple formazan crystals in viable cells by mitochondrial dehydrogenases.^38,56^ In this study, the absorbance values of the dissolved formazan crystals at a wavelength of 570 nm were measured by using the microplate reader. Table 5 summarizes the MTT assay results of the typical samples. In this study, three controls (blank: without samples, negative and positive controls) were used to verify consistency and accuracy from human errors, procedures, and conditions. The blank and negative control exhibited viable cell values close to 100%. For the positive control sample, the viable cell value was near to 0%. The results confirm the validity and reliability of the MTT assays used for the sample testing. As claimed by the ISO standard (ISO 10993-5), the sample is toxic when the death cell is more than 30%, which implies that the viability of extract-treated cells is less than 70% negative control (blank).^38,56^ Consequently, as shown in Table 5, for only g-LBA, the cell viability was less than 70%. When comparing with g-BBA, the cell viability was higher than that of the g-LBA. Obviously, it was found that the amount of sulfur trioxide (SO_3_) in LBA was higher than that of BBA (see Table 1). Different amounts of SO_3_ contained in g-LBA and g-BBA may be related to the differences of cell viability values. During the coal combustion, the sulfur (S) element in coals is oxidized into sulfur oxide (SO_2_), and only small amounts are further oxidized to SO_3_ in the boiler. If there is a selective catalytic reaction (SCR) technology for controlling NOx emission, some fraction of SO_2_ will be further oxidized to SO_3_ by the SCR catalyst.^57^ Notably, SO_3_ in the ashes is not present in a free form but predominantly exists as anhydrite (CaSO_4_), as evidenced by the XRD patterns of the coal ashes shown in Figure 5. During high-temperature sintering above approximately 1200 °C, anhydrite is decomposed and releases sulfur-containing gases (e.g., SO_2_). This decomposition is further accelerated in the presence of additives such as SiO_2_, Fe_2_O_3_, and Al_2_O_3_.^58^ Consistent with this behavior, the anhydrite phase disappears as seen in the XRD patterns of the sintered foams WF2 and WF3 (Figure 9) and WF4 and WF5 (Figure 11) after sintering at 1250 °C. Significantly, all the sintered foams had cell viability over 70%. This finding may indirectly imply that the sintering process could reduce the toxicity effect of g-LBA.

**Table 5:** MTT Assay Results of Typical Samples

	**Average values**	
**Samples**	**OD**_**570 nm**_	**Cell viability**(%)
**Control samples**		
1. Control No.1: Blank	1.034	100
2. Control No.2: Negative control	1.028	99
3. Control No.3: Positive control	0.000	0
**Powder samples**		
4. g-LBA	0.659	63
5. g-BBA	0.958	92
6. Mixed powder SiO_2_ (72 wt %) and Al_2_O_3_ (28 wt %)	0.987	95
**Foam samples**		
7. Foams: WF1 (1250 °C for 4 h)	0.956	92
8. Foams: WF2 (1250 °C for 4 h)	0.933	90
9. Foams: WF3 (1250 °C for 4 h)	0.915	88
10. Foams: WF4 (1250 °C for 4 h)	0.929	89
11. Foams: WF5 (1250 °C for 4 h)	0.997	96

## 4 Conclusions

This study demonstrates the feasibility of using coal bottom ashes (CBAs) from two distinct power plants as ceramic raw materials for fabricating ceramic foams owing to their high alumina (Al_2_O_3_) and silica (SiO_2_) content. XRF and XRD analyses revealed significant differences in oxide compositions and crystallinity phases between lignite bottom ash (LBA) and bituminous bottom ash (BBA). To achieve suitable particle size for the slurry preparation, lignite bottom ash (LBA) and bituminous bottom ash (BBA) were wet ground for 1 day and mixed with a commercial type of Al_2_O_3_ in 70:30 and 50:50 wt.% ratios. The resulting slurries were used for the foam fabrication and successfully sintered at 1250–1300 °C for LBA-derived foams and 1400–1450 °C for BBA-derived foams.

Experimental results indicate that the presence of CaO, Fe_2_O_3_, and SO_3_ in LBA and BBA plays a critical role in reducing the sintering temperature and determining foam color. Especially, LBA foams exhibited green-orange shade, whereas BBA foams showed red-orange-brown shade. The final color ranges were influenced by chemical compositions and sintering temperatures. Mechanical characterization results showed that both LBA- and BBA-derived foams possess densities and compressive strengths comparable to values reported in the literature. Cytotoxicity tests confirmed biocompatibility, with cell viability exceeding 70% for all fabricated foams. These findings highlight the potential of CBA not only as a sustainable raw material for ceramic foam production but also as a natural colorant for ceramic applications, promoting value-added utilization of coal bottom ashes in the ceramics industry.

## Supplementary Material



## Notes

Bulk density of fabricated foams were measured and discussed (PDF)
