# Supplementary material for: Sustainable Utilization of Lignite Bottom Ash (LBA) and Bituminous Bottom Ash (BBA) for Ceramic Foam Production
Source: ACS Omega. 2026 Apr 3;11(14):21605–17. doi: 10.1021/acsomega.5c06610 (PMC13084447; doi:10.1021/acsomega.5c06610)
Supplement: Supplementary file 1 [file ao5c06610_si_001.pdf]

Supporting Information

Sustainable utilization of lignite bottom ash (LBA) and bituminous bottom ash (BBA) for ceramic foam production

Wadwan Singhapong[1], Angkhana Jaroenworaluck[1,*], Watchara Chokevivat[1,*], Chutima Vanichvattanadecha[2]

[1]National Metal and Materials Technology Center (MTEC),

National Science and Technology Development Agency (NSTDA)

111 Thailand Science Park, Phahonyothin Road, Khlong Nueng, Khlong Luang,

Pathum Thani, 12120, Thailand

[2]National Nanotechnology Center (NANOTEC),

National Science and Technology Development Agency (NSTDA)

111 Thailand Science Park, Phahonyothin Road, Khlong Nueng, Khlong Luang,

Pathum Thani, 12120, Thailand

*Corresponding authors:

1. Angkhana Jaroenworaluck (ajangkhanaj@gmail.com)

2. Watchara Chokevivat (watcharc@mtec.or.th)

Tel.: +66 2 546 6500

Bulk Density of Fabricated Foams

The bulk densities and the apparent porosity of the sintered foams derived from LBA and BBA were measured using the liquid displacement method based on Archimedes' principle, in accordance with ASTM C373-88.[1] Figure S1 shows the average bulk densities and the apparent porosity of LBA derived foams sintered at 1250°C (WF2-1250 and WF3-1250) and BBA derived foams sintered at 1450°C (WF4-1450 and WF5-1450). The results indicate that the BBA derived foams exhibit slightly higher bulk densities than the LBA derived foams, although the difference is not obvious. The apparent porosity of the foams exhibits the similar trend as the bulk density. The bulk density of our fabricated foams are comparable to those 10 ppi alumina open-cell ceramic foams which possess a bulk density of 2.07 g/cm^3.[2]

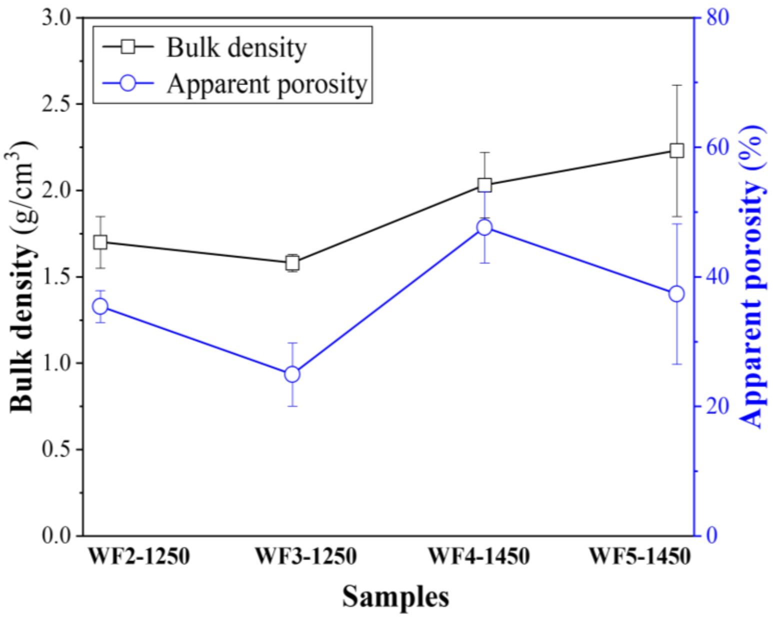

Figure S1. Bulk density and apparent porosity of the sintered foams determined by using Archimedes' method.

References

(1) ASTM International. Standard Test Method for Water Absorption, Bulk Density, Apparent Porosity, and Apparent Specific Gravity of Fired Whiteware Products. **2006**. DOI: 10.1520/C0373-88R06.

(2) Zhang, J.; Wu, H.; Dai, Y.; Voigt, C.; Gehre, P.; Jankovský, O.; Yan, W.; Li, Y.; Aneziris, C. G. Thermo-mechanical Behavior of Reticulated Alumina Foam Ceramics and the Influence of Structure Characteristics. *J. Eur. Ceram. Soc.* **2026**, *46*, 117746. DOI: 10.1016/j.jeurceramsoc.2025.117746.
